# Supplementary material for: Activation and Regulation of Pancreatic Stellate Cells in Chronic Pancreatic Fibrosis: A Potential Therapeutic Approach for Chronic Pancreatitis
Source: Biomedicines. 2024 Jan 4;12(1):108. doi: 10.3390/biomedicines12010108 (PMC10813475; doi:10.3390/biomedicines12010108)
Supplement: Supplementary file 1 [file biomedicines-12-00108-s001.zip › biomedicines-2754978-supplementary.pdf]

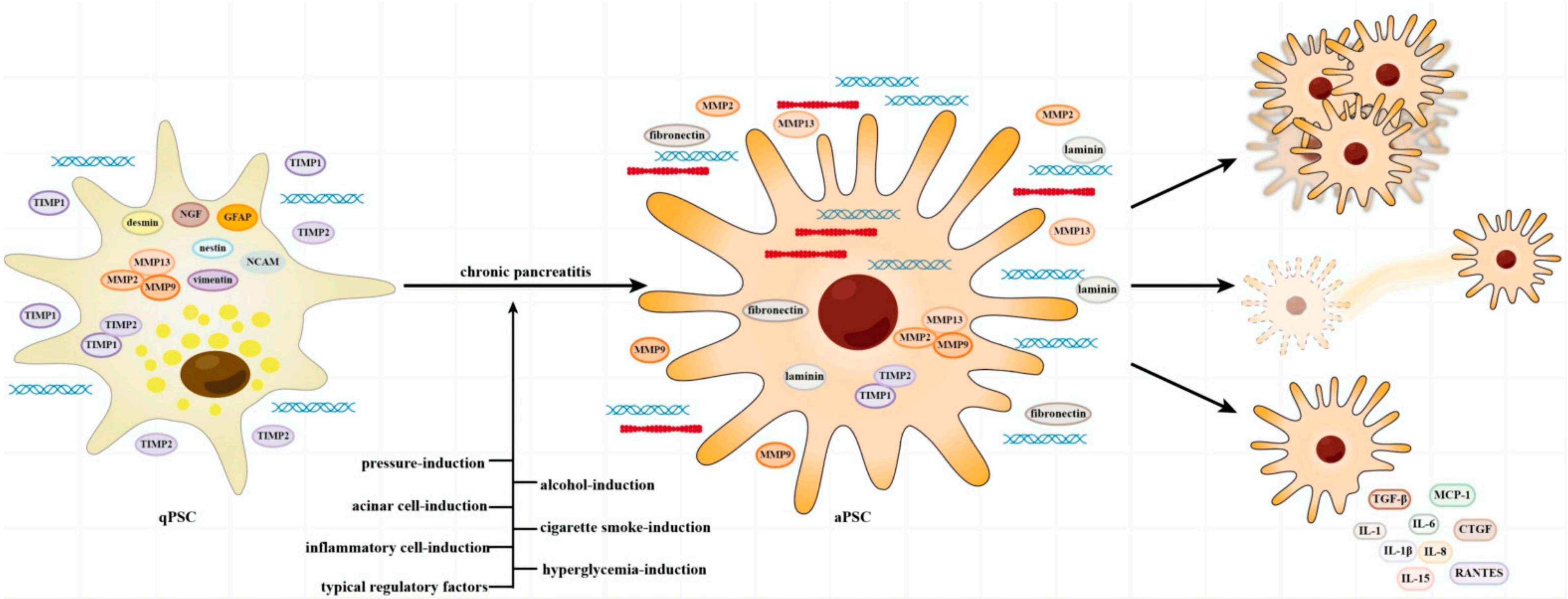

Figure S1. This figure illustrates the molecular and cellular characteristics of pancreatic stellate cells before and after activation.
